# Whole genome sequencing and analysis of Swarna, a widely cultivated *indica* rice variety with low glycemic index

**DOI:** 10.1038/srep11303

**Published:** 2015-06-11

**Authors:** Pasupathi Rathinasabapathi, Natarajan Purushothaman, Ramprasad VL, Madasamy Parani

**Affiliations:** 1 Genomics Laboratory, Department of Genetic Engineering, SRM University, Chennai, Tamil Nadu- 603 203, India; 2 SciGenom, Kakkanad, Cochin, Kerala – 682037, India

## Abstract

Swarna is a popular cultivated *indica* rice variety with low glycemic index (GI) but its genetic basis is not known. The whole genome of Swarna was sequenced using Illumina’s paired-end technology, and the reads were mapped to the Nipponbare reference genome. Overall, 65,984 non-synonymous SNPs were identified in 20,350 genes, and *in silico* analysis predicted that 4,847 of them in 2,214 genes may have deleterious effect on protein functions. Polymorphisms were found in all the starch biosynthesis genes, except the gene for branching enzyme IIa. It was found that T/G SNP at position 246, ‘A’ at position 2,386, and ‘C’ at position 3,378 in the granule bound starch synthase I gene, and C/T SNP at position 1,188 in the glucose-6-phosphate translocator gene may contribute to the low GI phenotype in Swarna. All these variants were also found in the genome of another low GI indica rice variety from Columbia, Fedearroz 50. The whole genome analysis of Swarna helped to understand the genetic basis of GI in rice, which is a complex trait involving multiple factors.

Rice is the primary source of dietary energy for half of the World population, and 90% of them reside in low and middle income countries in Asia. In recent years, rice consumption is steadily increasing outside Asia also, especially in sub-Saharan African regions (www.iriri.org). The regular consumption of rice as a primary food increases the risk of developing type 2 diabetes by elevating the postprandial glucose level, a condition referred to as postprandial hyperglycemia (PPHG). PPHG stimulates deleterious structural changes in many tissues in the body^1^, and increases the risk for cardiovascular diseases in diabetic as well as non-diabetic people^2^. PPHG due to the consumption of rice is caused by the rapid breakdown down of its carbohydrates to glucose. Propensity of the food to raise the blood glucose level after consumption is measured or estimated as glycemic index (GI). Foods with lower GI are preferred to keep PPHG under control because the carbohydrates in them do not rapidly breakdown into glucose.

GI of rice varieties is highly dependent on the composition of the carbohydrates in the seeds. In general, *indica* rice varieties have lower GI than *japonica* rice varieties^3^. A recent study reported that Swarna, an *indica* rice variety from India, was having low GI^4^. This variety was developed in the year 1982 by crossing Vasista and Mahsuri rice varieties, and it is popularly cultivated in the southern states of India. Understanding the genetic basis of low GI in Swarna will help in breeding this trait in other rice varieties. Natural variations in the 18 starch biosynthesis related genes contribute directly or indirectly to the GI by altering the amylose and amylopectin content in rice^5^. Availability of the reference genome sequences makes it easier to study the genome-level genetic variations in rice.

Whole genome sequencing using next-generation sequencing (NGS) technologies could identify millions of variants in a rapid, efficient, high throughput, and cost effective manner. These variants have been used as DNA markers that aid in marker-assisted breeding and quantitative trait locus analysis^6^,^7^. He *et al.* (2011)^8^ have identified the genomic regions that influenced the domestication of rice by mapping the genome of *O. sativa indica*, *O. sativa japonica* and *O. rufipogon*. The breeding history of 20 rice varieties was revealed by comparing the genome sequence of landraces and modern rice varieties^9^. Deep whole genome re-sequencing was carried out to identify genome-wide polymorphisms in a widely used rice maintainer line (V20B), and 7 cultivated varieties of temperate and tropical *japonica* groups^10^,^11^.

To our knowledge, there has been no whole genome-based polymorphism study on rice varieties with low GI. Therefore, we have sequenced the whole genome of Swarna rice variety by Illumina’s paired-end sequencing, and identified single nucleotide polymorphisms (SNPs), insertions and deletions (InDels) by aligning it with the Nipponbare genome sequence as reference. In addition to the detailed analysis of the 18 starch biosynthesis related genes, an account of reference assembled reads as well as *de novo* assembled reads has also been provided. The results from this study will help in understanding the genetic basis of GI, and its utilization in developing rice varieties with anti-diabetic properties.

## Results

### Whole Genome Sequencing and Assembly

**Figure 1:**
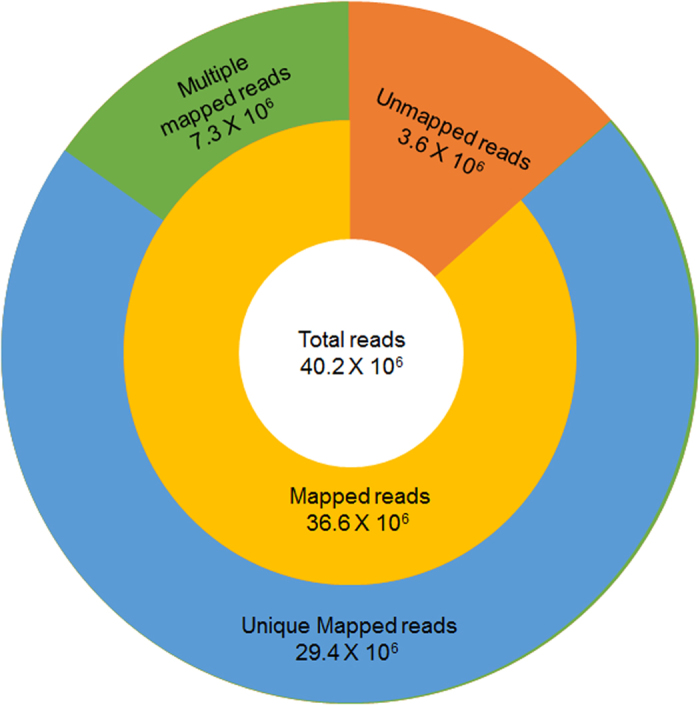
Classification of the Swarna reads mapped on to the Nipponbare reference genome. The total number reads in the center circle. Number of mapped reads on the nuclear genome and unmapped reads in the middle circle. The outer circle represents the uniquely mapped reads, and the reads mapped to multiple locations.

Sequencing the genome of Swarna rice variety using Illumina Hiseq 2500 platform yielded 4.2 GB of high quality sequence data. Totally 40.2 × 10^6^ reads with an average read length of 101 bp were obtained, and the average sequencing depth was 10×. We mapped 36.6 × 10^6^ (91.0%) reads to the *Oryza sativa* L. cv. Nipponbare reference genome, and the unmapped reads were *de novo* assembled (described later). Out of the mapped reads, 80.3% (29.4 × 10^6^) reads were uniquely mapped to the chromosomes, and the remaining reads were mapped to multiple locations on the reference genome (Fig. 1). Sequence coverage of the uniquely mapped reads ranged between 75.9% (chromosome 11) and 89.9% (chromosome 3) with an average of 82.8% (Table 1). Only the uniquely mapped reads were used for the purpose of identifying the SNPs and InDels. The BAM file generated for this study was submitted to the sequence read archive (SRA) at NCBI with the accession number PRJNA270390.

### Variant Identification

The SNPs and InDels in *O. sativa* L. cv. Swarna were determined with reference to the Nipponbare reference genome. Initially 3,400,394 variants (3,092,345 SNPs and 308,049 InDels) were identified using default parameters. Application of the filtering criteria yielded 1,253,861 quality filtered variants, which included 1,149,698 SNPs and 104,163 InDels (49,680 insertions and 54,483 deletions). The ratio of homozygous to heterozygous variants was 27.4 for SNPs and 53.7 for InDels. Total number of variants was found to be the highest in chromosome 1 (151,124), and the lowest in chromosome 9 (78,817). Average number of variants per chromosome was 104,488 at the rate of one variant for every 297 bases. Genomic distribution of SNPs and InDels was determined by calculating the density of occurrence (see Supplementary Fig.S1 & Supplementary Fig.S2 online). SNP density was the highest in chromosome 10 (351.7), and the lowest in chromosome 4 (256.3). Similarly, InDels density was the highest in chromosome 2 (30.6), and the lowest in chromosome 4 (22.1) (Table 2). Average density of variants across the whole genome was 307.3 SNPs and 27.6 InDels. We have identified 352 high SNP density regions (SNP/kb > 5) and 157 low SNP density regions (SNP/kb < 0.1). Some chromosomes had more number of high density SNP regions while others had more number of low SNP density regions. For example, while chromosome 11 had 44 high density and 3 low density regions, chromosome 5 had 35 low density and 20 high density regions (Table 3). The genome of Swarna contained 38 successive high density regions and 29 successive low density regions of 0.2 to 3.4 Mb. The longest high density region (0.4 Mb) was found in chromosome 11, and the longest low density region (3.4 Mb) was found in chromosome 5. Regarding the InDels, only low density regions were detected in all the chromosomes except chromosome 12, which had low as well as high density regions. Copy number variation (CNV) analysis showed the presence of 6,732 structural variations, which included 4,039 deletions, 381 inversions, and 1,988 translocations.

### Annotation of Variants

**Figure 2:**
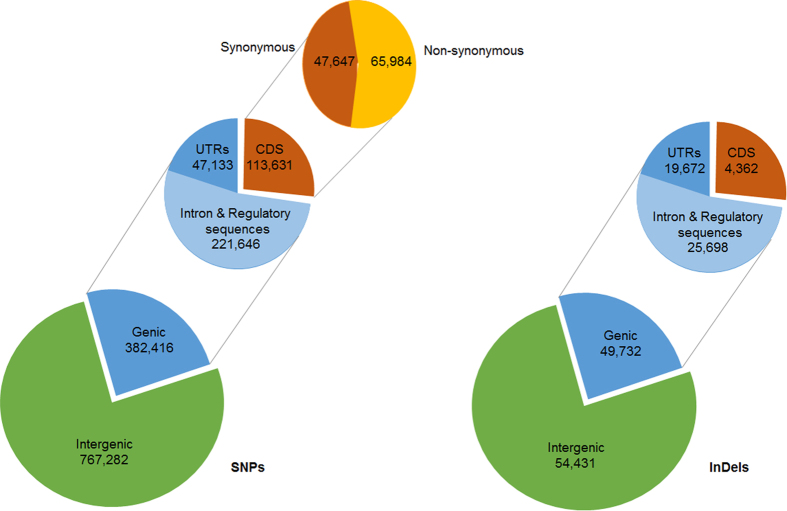
Annotation of SNPs and InDels that were identified in Swarna. Annotated SNPs and InDels were classified based on their locations.

**Figure 3:**
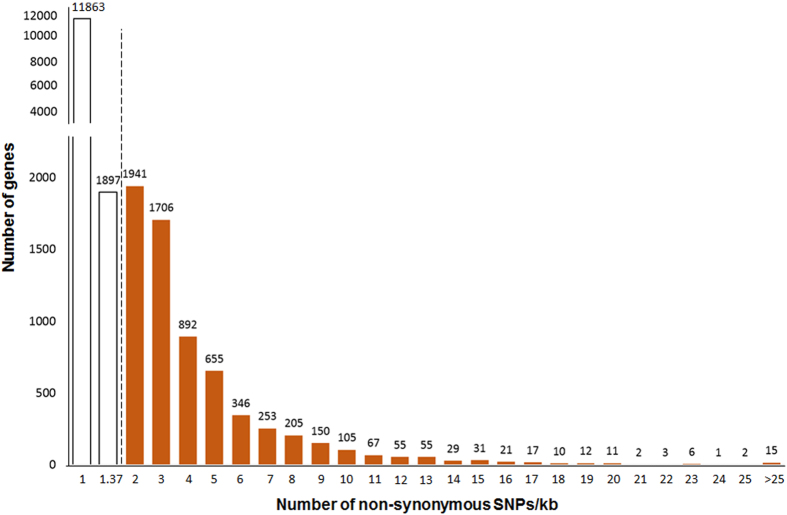
Distribution and skewness of the number of non-synonymous SNPs per kb in the 20,350 genes of Swarna. The outlier value calculation indicated that 6,589 genes had >1.37 non-synonymous SNPs per kb (orange bars).

Among the 1,149,698 SNPs that were found in Swarna, 382,416 and 767,282 SNPs were in genic and intergenic region, respectively (Fig. 2). The ratio of transition to transversion SNPs ranged between 2.07 (chromosome 12) and 2.28 (chromosome 5) with an average of 2.2. It was found that 65,984 SNPs in the genic region were non-synonymous SNPs. These SNPs were spread over 20,350 genes, and the number of non-synonymous SNPs/gene varied between 1 and 116. The number of non-synonymous SNPs/kb of genic region ranged between 0.03 and 41.59. Five number summary of the box-and-whisker plot was used to calculate the outlier value (1.37), and 6,590 genes were classified as outliers as they had >1.37 non-synonymous SNPs/kb of genic region (Fig. 3).Among the 104,163 InDels that were found in Swarna, 49,732 and 54,431 InDels were detected in the genic and intergenic regions, respectively. It was observed that 4,362 InDels were detected in the coding regions (Fig. 2). The length of the InDels ranged from −39 to +27 bp, and majority of them were mono-nucleotide (32.49%) or di-nucleotide InDels (21.32%) (see Supplementary Fig.S3 online).

### Functional Effect of Non-synonymous SNPs

SIFT analysis predicted that 4,847 non-synonymous SNPs in Swarna may have deleterious effects on the protein functions (tolerance index < 0.05). These deleterious SNPs were located in 2,214 genes, and their number varied between 1 and 12 per gene. Among the 4,847 deleterious SNPs, 505 SNPs were predicted to be highly deleterious (tolerance index = 0.0). These highly deleterious SNPs were located in 452 genes, and their number varied between 1 and 4 per gene.

### Analysis of the Variants in the Starch Biosynthesis Related Genes (SSRGs)

The 18 SSRGs in Swarna contained 434 SNPs and 41 InDels. By comparing these SNPs to the OryzaSNP MSU database (http://oryzasnp.plantbiology.msu.edu/), 315 novel SNPs were identified in Swarna. Majority of the SNPs (403) and all the InDels were in introns and other non-coding regions. Coding regions contained 33 non-synonymous SNPs, which were validated by Sanger sequencing (see Supplementary Fig.S4 online). These non-synonymous SNPs were located in 12 SSRGs with a minimum of 1 (GPT1, SSIIa and ISA1), and a maximum of 10 (GBSSII) per gene. The proportion of non-synonymous (Ka) to synonymous SNP (Ks) is indicative of neutral (Ka/Ks = 1), negative (Ka/Ks < 1) or positive (Ka/Ks > 1) selection of the genes. The Ka/Ks ratio could not be calculated for 3 SSRGs, which did not have Ka, Ks or both. The Ka/Ks ratio revealed that 1, 10 and 4 SSRGs are under in neutral, negative and positive selection, respectively (Table 4).

The SIFT, SNAP and Provean algorithms predicted that 1, 2 and 3 non-synonymous SNPs, respectively, in the SSRGs may be deleterious to the protein functions. However, only one SNP in the starch synthase IIIb gene was predicted to be deleterious by all the three algorithms. I-Mutant predicted that 30 non-synonymous SNPs may reduce the protein stability, and 10 non-synonymous SNPs (DDG value > −1.0) may severely affect the protein stability.

### Annotation of Unmapped Reads

**Figure 4:**
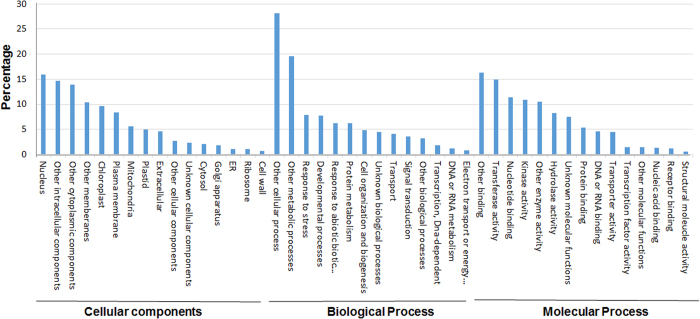
Functional categorization of the contigs assembled from the unmapped reads into gene ontology (GO) terms: biological processes, cellular components, molecular functions.

*De novo* assembly of the 3.6 × 10^6^ unmapped reads yielded 2,392 contigs that ranged in size between 500 and 6,188 bp with an average of 769 bp. In BLASTn analysis, 1,784 contigs did not show significant similarity with any sequence in the non-redundant nucleotide database at NCBI. Among the remaining 608 contigs that showed similarity with the sequences in the database, 504 (83%) contigs were closely related to the sequences from Oryza species. Gene Ontology (GO) analysis of the annotated contigs related them to cellular components, biological and molecular processes (Fig. 4). In Molecular processes, the highest number of contigs were related to the genes involved in binding to other macromolecules (40.2%) followed by transferase activity (14.9%) and kinase activity (10.8%). In cellular components, 54.9% of the contigs were related to nucleus, cytoplasmic, intracellular and membrane components. Majority of the contigs in biological processes were accounted for cellular (28.2%) and metabolic (19.6%) processes.

## Discussion

Development of rice varieties with low glycemic index (GI) would be desirable because regular consumption of normal rice is associated with 11% increase in the risk of developing type 2 diabetes^12^. In addition, dietary GI is positively associated with several metabolic risk factors^13^. A recent study from the International Rice Research Institute, Philippines, has identified Swarna, a cultivated *indica* rice variety, to have high amylose content and low GI of 60^4^. However, there was no further study to understand the genetic basis of these traits. The current study reports sequencing and analysis of the whole genome of Swarna, with special reference to the starch biosynthesis related genes (SSRGs). The genome of Swarna was mapped to *Oryza sativa* L. cv. Nipponbare, which in addition to be a standard reference genome, is a high GI rice variety with predicted GI of 85^14^. About 40 million 101 bp reads from Swarna were mapped to the Nipponbare genome. Though a large number of reads were uniquely mapped, mapping to multiple locations was also observed. About 11% of the reads did not map to the reference genome, which is comparable to the proportion of unmapped reads from IR64, another indica rice variety^15^. Failure of the reads to map to the reference genome could be due to the inherent genomic differences between *indica* and *japonica* subspecies^16^. Therefore, we have *de novo* assembled the unmapped reads to contigs, and analyzed them by BLASTn search against the nucleotide database at NCBI. A large number of the *de novo* assembled contigs did not show significant similarity with any sequence in the database, indicating their uniqueness to the genome of Swarna. Annotation of the remaining contigs has revealed that the sequences were homologous to the genes related to different cellular, biological and molecular functions in *Oryza*.

Analysis of the mapped reads showed that the distribution of the SNPs in Swarna was not even, and there were many continuous regions with poor number of SNPs, called ‘SNP deserts’. ‘SNP deserts’ were reported in genome mapping of *japonica* to *indica* as well as *indica* to *indica*^10^,^17^. We detected a large ‘SNP desert’ in chromosome 5 between 9.6 and 13.0 Mb with 0.098 SNP/kb. Others have detected a much bigger ‘SNP desert’ in the same region^10^,^18^. We identified two additional ‘SNP deserts’ of 1.7 Mb in chromosome 4 (25.4 to 27.1 Mb, 0.402 SNP/kb) and 1.0 Mb in chromosome 8 (23.7 to 24.7 Mb, 0.162 SNP/ kb), which were not reported from other rice genomes. The SNP deserts may represent pre-domestication bottleneck and human selection on the adaptive genes during crop improvement^19^.

We have observed more transition SNPs than transversion SNPs in Swarna. This kind of ‘transition bias’ in rice genomes was reported before, and the ratio of transition SNPs to transversion SNPs varied narrowly between 2.0 and 2.53^10^,^18^,^20^. The higher frequency of transitional SNPs over transversion SNPs is more likely to conserve the protein structures to preserve conformational advantages in natural selection^21^. Within the transition SNPs, the frequency of C/T SNPs was more than A/G SNPs, which may be attributed to the increased possibility of C/T mutation due to the spontaneous deamination of methylated cytosine residues^22^. The abundance of C/T SNPs over A/G SNPs was reported before in monocots as well as dicots^18^,^23^,^24^. Within transversion SNPs, the frequency of T/A SNPs was higher than A/C, G/T, and C/G SNPs in Swarna, as reported for the other rice genomes^15^,^18^.

A large number of non-synonyms SNP were found in Swarna but the major interest was to identify the SNPs that may affect the function of the proteins. Amino acid residues or motifs that are essential for the biological functions and stability of the proteins are likely to be more conserved than the others. Therefore, non-synonymous SNPs in the highly conserved regions may have profound effect on the protein functions. SIFT algorithm uses this phenomenon, and predicts the effect of non-synonymous SNPs as ‘tolerated’ or ‘deleterious’^25^,^26^. SIFT analysis of the non-synonymous SNPs predicted that 4,847 SNPs in 2,214 genes of Swarna may have deleterious effect on the proteins. Comparative information on deleterious non-synonymous SNPs in other rice genomes is not available.

The major carbohydrate in the rice seeds is starch, which is made of linear amylose and branched amylopectin. Digestive enzymes break down amylose more slowly than amylopectin. In addition, though retrogradation does not solely depend on starch content or quality^27^, amylose content does have significant effect on starch retrogradation^28^. Cooked rice with high amylose content is easily retrograded, which makes it harder for the digestive enzymes to act upon^29^. It was recently established that amylose content has negative correlation with GI^4^. Therefore, starch biosynthesis pathway that favors the synthesis of amylose over amylopectin would be desirable. ADP-glucose pyrophosphorylase (AGPase), granule bound starch synthase (GBSS), starch synthase (SS), branching enzyme (BE), debranching enzyme (DBE), starch phosphorylase (PHO), and glucose 6-phosphate translocator (GPT) are the seven enzyme classes encoded by at least 18 SSRGs, which are involved in starch biosynthesis in rice^30^. Association studies in a population of 233 japonica-type F_6_ rice breeding lines have identified SNPs in the GPT1 and GBSSI genes to be highly associated with high amylose content and retrogradation (*F* value: 123 and 121 for amylose content, 292 and 223 for retrogradation)^5^.

Analysis of the variants in the 18 SSRGs in Swarna revealed the complete absence of variants in PUL and BEIIa genes. Manual verification using the genomic coordinates confirmed the absence of variants in BEIIa gene. Genomic coordinates for the PUL gene could not be obtained from the MSU7 rice genome database (http://rice.plantbiology.msu.edu/), and we found that this gene was not annotated in the reference genome itself. Available mRNA sequence for the PUL gene from Nipponbare (Accession No. NM_001058664.1) contained only partial coding sequence (CDS). Therefore, we have used a 100% identical complete CDS from Taihunuo (a *japonica* rice cultivar, Accession No. GQ150890.1), and manually marked the genomic coordinates. Manual annotation of the variants revealed that the PUL gene in Swarna actually contained 52 variants. Therefore, it was found that all the SSRGs, except BEIIa gene, were polymorphic in Swarna. Roth *et al.* (2006)^31^ have used the ratio of non-synonymous to synonymous SNPs (Ka/Ks ratio) for predicting the nature of selection on individual genes. Using the same criteria, the SSIIa gene was found to be under neutral conditions of evolution in *indica* (this study) as well as *japonica* rice^32^. The other SSRGs were found to be still evolving under positive or negative selection.

Among the 475 variants that were identified in the SSRGs, 404 were in non-coding regions, and 71 were in the coding regions. One of the non-coding SNPs, T/G at position 246, was present at the intron/exon 1 junction site of GBSSI gene. Presence of ‘T’ in this position causes aberrant splicing of the first intron, and the aberrantly spliced mRNAs are not translated to GBSS1 protein. As a result, the normal and aberrantly spliced mRNAs were produced in the ratio of 1:5, and the amylose content was reduced^33^. In Swarna, homozygous ‘G’ was present in this position, which entailed normal splicing of the GBSSI mRNAs and high amylose content. Among the 71 coding SNPs, a C/T non-synonymous SNP at position 1,188 in GPT1 gene, which alters Leu42 to Phe was present in homozygous condition. This SNP was reported to be strongly associated with high amylose content and retrogradation in rice^5^. Combination of these two SNPs, in homozygous condition, was reported to be associated with highest amylose content and retrograded resistant starch in rice^34^. The GBSSI gene in Swarna also had ‘A’ at position 2,386 and ‘C’ at position 3,378, a two-nucleotide combination, which was reported to favor high amylose phenotype in rice^35^. These nucleotides were not flagged as SNPs in Swarna, because the reference genome had the same nucleotides at the respective positions. This observation highlights that analysis of the variants in the genes of interest requires further study besides automated SNP calling. All the above described variants were found in Fedearroz 50, which is a low GI indica rice variety from Columbia (http://oryzasnp.org/iric-portal).

*In silico* investigation of the functional impact of the 33 non-synonymous revealed that the T/C SNP at position 6,422 in the SSIIIb gene (Glu643Gly) may be deleterious and would reduce the protein stability. Though lack of expression of starch synthase can increase the amylose content at the cost of chain elongation in amylopectin^36^, SSIIIb gene is particularly expressed only in leaves^37^,^38^; hence may not directly affect the amylose content in seeds. Further, the C/T SNP at position 1,188 in the GPT1 gene (Leu42Phe) was among the remaining 32 non-synonymous that were predicted to have no deleterious effect on protein function. This SNP was reported to be associated with high amylose content^5^, probably due to its positive effect on GPT1. However, role of this SNP on amylose content could not be clarified in the absence of a genotype with C/T at position 1,188 in GPT1 gene without T/G at position 246 in GBSSI gene. Also more reliable prediction from *in silico* analysis should wait for the availability of the crystal structures for these proteins.

## Methods

### Sample Collection, DNA Isolation and Genome Sequencing

Seeds of *Oryza sativa* L. cv. Swarna were obtained from Tamil Nadu Agricultural University, Thirurkuppam, Tamil Nadu, India. The seeds were germinated under the controlled environment, and genomic DNA was isolated from the young leaves using cetyl trimethyl ammonium bromide (CTAB) method^39^. Whole genome sequencing was done using Illumina’s paired-end sequencing technology on Hiseq 2500 systems (Illumina Inc., USA). A paired-end library was prepared from the genomic DNA as per the manufacturer’s protocol (Illumina Inc., USA). Clonal DNA clusters were generated using cBOT and TruSeq PE Cluster kit v3-cBot-HS (Illumina Inc., USA). TruSeq SBS v3-HS kit (Illumina Inc., USA) was used to sequence DNA of each cluster on a flow cell to generate 100 bp paired-end reads. The reads were extracted as paired-end reads in FASTQ format for further downstream analysis. Validation of SNPs was done by Sanger sequencing of the PCR products, which were amplified using gene-specific primers.

### Variant Identification and Annotation

The Illumina TruSeq DNA adapters in the paired-end reads were trimmed using ‘cutadapt’ (https://code.google.com/p/cutadapt/). The trimmed reads were filtered using sickle master (https://github.com/najoshi/sickle) by retaining the bases with minimum Phred quality score of 30, and truncation of the sequences with Ns. The quality filtered paired-end reads were aligned to the *Oryza sativa* L. cv. Nipponbare reference genome, the unified build release Os-Nipponbare-Reference-IRGSP-1.0 (IRGSP-1.0)^40^ using Burrows Wheeler alignment (BWA) tool^41^ with default parameters. The reads were also mapped to the chloroplast genome (DDBJ Acc. No. X15901) and the mitochondrial genome (DDBJ Acc. No. DQ167400) using BWA tool. The aligned reads in the SAM file were sorted using SortSam of Picard tool V1.118, and the sorted SAM file was converted to BAM file for variant calling using SAMtools V0.1.19. Variant calling was performed using ‘mpileup’ of SAMtools^42^ with default parameters to identify SNPs and InDels. In order to minimize the rate of false-positive SNPs, the variant calling file (VCF) was filtered based on (1) number of reads per base between 5 and 75, (2) base quality ≥ 30, (3) mapping quality ≥ 60, (4) variant quality ≥ 90, and (5) distance of adjacent variant ≥ 5. The filtered variants were annotated using the rice7 gene model database for *Oryza sativa* (http://sourceforge.net/projects/snpeff/files/databases/v3_6/ snpEff_v3_6_rice7.zip) and SnpEff V3.6 tool^43^. SNPs and InDels in the genes and other genomic regions were annotated as genic and intergenic, respectively. The SNPs were segregated as transition (C/T and G/A) and transversion (C/G, T/A, A/C and G/T) SNPs. The SNPs and InDels in the genic regions were further classified based on their location in exons, introns, 5’UTRs, 3’UTRs, coding regions, and splice-site regions. The SNPs in coding regions were segregated as synonymous (cause no change in amino acid), non-synonymous (cause change in amino acid), stop gain (introduce a stop codon), stop loss (remove existing stop codon), start gain (introduce a start codon), and start loss (remove existing start codon).

Densities of SNPs and InDels in every 100 kb region were calculated for locating hyper- and hypo-varying regions. The chromosome regions were defined as hyper-varying regions if SNPs/kb > 5 or InDels/kb > 1, and hypo-varying regions if SNPs/kb < 0.1 or InDels/kb < 0.01. Copy number variation (CNV) analysis was done using GASV software^44^ (https://code.google.com/p/gasv/).

### Prediction of Deleterious Non-synonymous SNP

The impact of amino acid substitution on protein function and phenotype alteration were studied by using SIFT algorithm (http://sift.jcvi.org), which classifies the effect of the SNP as ‘tolerated’ or ‘deleterious’. The SIFT algorithm generates alignments with a large number of homologous sequences and assigns a tolerance index value of 0 to 1 to each residue. The SNPs are classified as tolerated (tolerance index > 0.05) or deleterious (tolerance index < 0.05)

### Analysis of the Variants in Starch Biosynthesis Related Genes (SSRGs)

The functional effect of the non-synonymous SNPs in the 18 SSRGs were analyzed by using SIFT (http://sift.jcvi.org) Provean (http://provean.jcvi.org) and SNAP (https://rostlab.org/services/snap/). A non-synonyms SNP was designated as deleterious only if all the three algorithms categorized it as deleterious SNP. I-Mutant (version 2.0) (http://folding.uib.es/i-mutant/i-mutant2.0.html), a neural network based tool, was used to predict the effect of the non-synonymous SNPs on protein stability. I-Mutant estimates free energy change value (difference in ΔG, DDG), which is the difference in Gibb’s free energy value of the mutated and native protein (kcal/mole). Based on the value of DDG, the SNPS were predicted to decrease (DDG < 0) or increase (DDG > 0) the protein stability.

### *De novo* Assembly and Annotation of the Unmapped Reads

The unmapped reads from the BWA-MEM alignment file were extracted in to a BAM file using SAMtools^42^. The reads in the BAM file were converted to FASTQ format using BAMtools V2.3.0. The unmapped FASTQ reads were assembled *de novo* using Velvet V1.2.10^45^. The assembly was optimized using different Kmer sizes, and contigs with more than 500 bp that were used for further analysis. The *de novo* assembled contigs were annotated using BLASTn algorithm at NCBI (http://blast.ncbi.nlm.nih.gov/Blast.cgi) against non-redundant nucleotide database. Functional classification of the contigs was done using Gene Ontology (GO) tool at TAIR (https://www.arabidopsis.org/). The contigs were classified under three GO categories, such as biological processes, molecular functions and cellular components.

## Additional Information

**How to cite this article**: Rathinasabapathi, P. *et al.* Whole genome sequencing and analysis of Swarna, a widely cultivated *indica* rice variety with low glycemic index. *Sci. Rep.* **5**, 11303; doi: 10.1038/srep11303 (2015).

## Supplementary Material

Supplementary Information

## Figures and Tables

**Table 1:** Mapping and coverage of the reads from Swarna to the Nipponbare reference genome IRGSP1.0.

**Chromosome**	**Nipponbare genome (bp)**	**Uniquely covered bases**	**Coverage (%)**	**Uniquely covered reads**
1	43,270,923	37,267,274	86.1	3,446,257
2	35,937,250	31,252,011	87.0	2,906,369
3	36,413,819	32,749,423	89.9	3,037,514
4	35,502,694	28,162,153	79.3	2,665,489
5	29,958,434	26,207,749	87.5	2,463,462
6	31,248,787	25,988,755	83.2	2,454,467
7	29,697,621	24,291,613	81.8	2,262,245
8	28,443,022	23,369,142	82.2	2,199,600
9	23,012,720	18,969,755	82.4	1,792,075
10	23,207,287	19,040,419	82.0	1,970,091
11	29,021,106	22,012,814	75.9	2,035,231
12	27,531,856	21,029,590	76.4	2,134,701
Total/ Average	373,245,519	310,340,698	82.8	29,367,501

**Table 2:** Number and densities of SNPs and InDels in the genome of Swarna.

**Chromosome**	**SNPs**		**InDels**	
	**Number**	**Density**	**Number**	**Density**
1	138,059	318.4	13,065	29.9
2	119,105	330.4	11,107	30.6
3	114,760	314.0	11,148	30.4
4	91,355	256.3	7,907	22.1
5	81,585	272.4	7,738	25.7
6	99,433	317.3	9,152	29.0
7	90,566	297.0	8,141	27.2
8	83,665	293.2	7,507	26.1
9	72,574	315.1	6,243	26.9
10	81,711	351.7	7,022	30.1
11	98,730	338.7	8,396	28.7
12	78,155	282.8	6,737	24.2
Total/Average	1,149,698	307.3	104,163	27.6

**Table 3:** Number of high and low SNP and InDel density regions.

**Chromosome**	**SNP density**		**InDel density**	
	**High**	**Low**	**High**	**Low**
1	37	17	0	10
2	29	8	0	8
3	26	6	0	9
4	21	21	0	18
5	20	35	0	31
6	25	17	0	17
7	36	16	0	18
8	28	10	0	11
9	26	9	0	7
10	32	2	0	3
11	44	3	0	5
12	28	13	1	13
Total	352	157	1	150

**Table 4:** Details of the SNPs and InDels that were identified in the starch biosynthesis related genes (SSRG) of Swarna.

**S.No**	**Gene Name**	**Gene Symbol**	**Non-Coding SNPs**	**Non-Synonymous SNPs (Ka)**	**Synonymous SNPs (Ks)**	**Ka/Ks**	**Non-coding InDels**	**Coding InDels**
1	ADP- glucose pyrophosphorylase (small unit)	AGPS2b	14	0	2	0.0	1	0
2	Alpha 1,4- glucan phosphorylase	SPHOL	9	0	1	0.0	1	0
3	Glucose 6-phosphate-translocator	GPT1	9	1	2	0.5	2	0
4	Granule-bound starch synthase I	GBSSI	10	0	0	–	4	0
5	Granule-bound starch synthase II	GBSSII	82	10	2	5.0	7	0
6	Starch synthase I	SSI	59	2	7	0.3	7	0
7	Starch synthase IIa	SSIIa	16	1	1	1.0	0	0
8	Starch synthase IIb	SSIIb	14	3	1	3.0	3	0
9	Starch synthase IIIa	SSIIIa	20	3	4	0.8	2	0
10	Starch synthase IIIb	SSIIIb	13	4	6	0.7	3	0
11	Starch synthase IVa	SSIVa	11	2	1	2.0	1	0
12	Starch synthase IVb	SSIVb	17	0	3	0.0	0	0
13	Branching enzyme I	BEI	9	0	2	0.0	1	0
14	Branching enzyme IIa	BEIIa	0	0	0	—	0	0
15	Branching enzyme IIb	BEIIb	23	2	3	0.7	5	0
16	Debranching enzyme -isoamylase 1	ISA1	9	1	2	0.5	1	0
17	Debranching enzyme -isoamylase 2	ISA2	1	2	1	2.0	0	0
18	Debranching enzyme -Pullulanase	PUL	47	2	0	—	3	0
	Total/ average		363	33	38	0.9	41	0
