# Supplementary material for: Whole genome sequencing and analysis of Swarna, a widely cultivated indica rice variety with low glycemic index
Source: Sci Rep. 2015 Jun 11;5:11303. doi: 10.1038/srep11303 (PMC4464077; doi:10.1038/srep11303)
Supplement: Supplementary Information [file srep11303-s1.pdf]

Whole genome sequencing and analysis of Swarna, a widely cultivated *indica* rice variety with low glycemic index

Pasupathi Rathinasabapathi[1], Natarajan Purushothaman[1], Ramprasad VL[2], Madasamy Parani[1]*

[1] Genomics Laboratory, Department of Genetic Engineering, SRM University, Chennai, Tamil Nadu- 603 203, India.

[2] SciGenom, Kakkanad, Cochin, Kerala – 682037, India.

*Corresponding author: Madasamy Parani Email: parani.m@ktr.srmuniv.ac.in

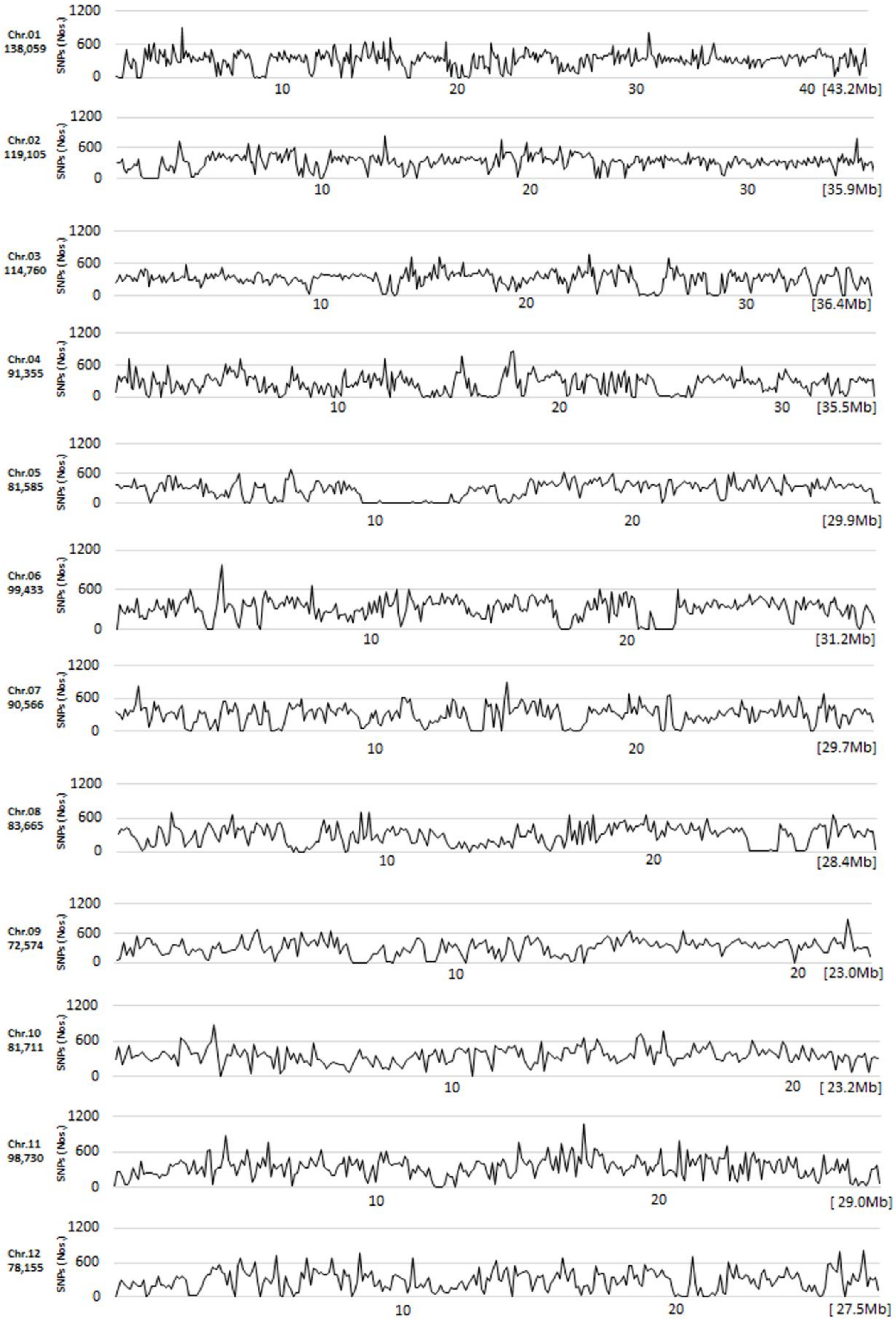

Supplementary Fig. S1: Distribution of SNPs detected in Swarna in each chromosome. The x-axis represented the physical distance along each chromosome, split into 100 kb windows. The total size of each chromosome was shown in brackets. The y-axis indicated the number of SNPs. The total number of SNPs in each chromosome was mentioned.

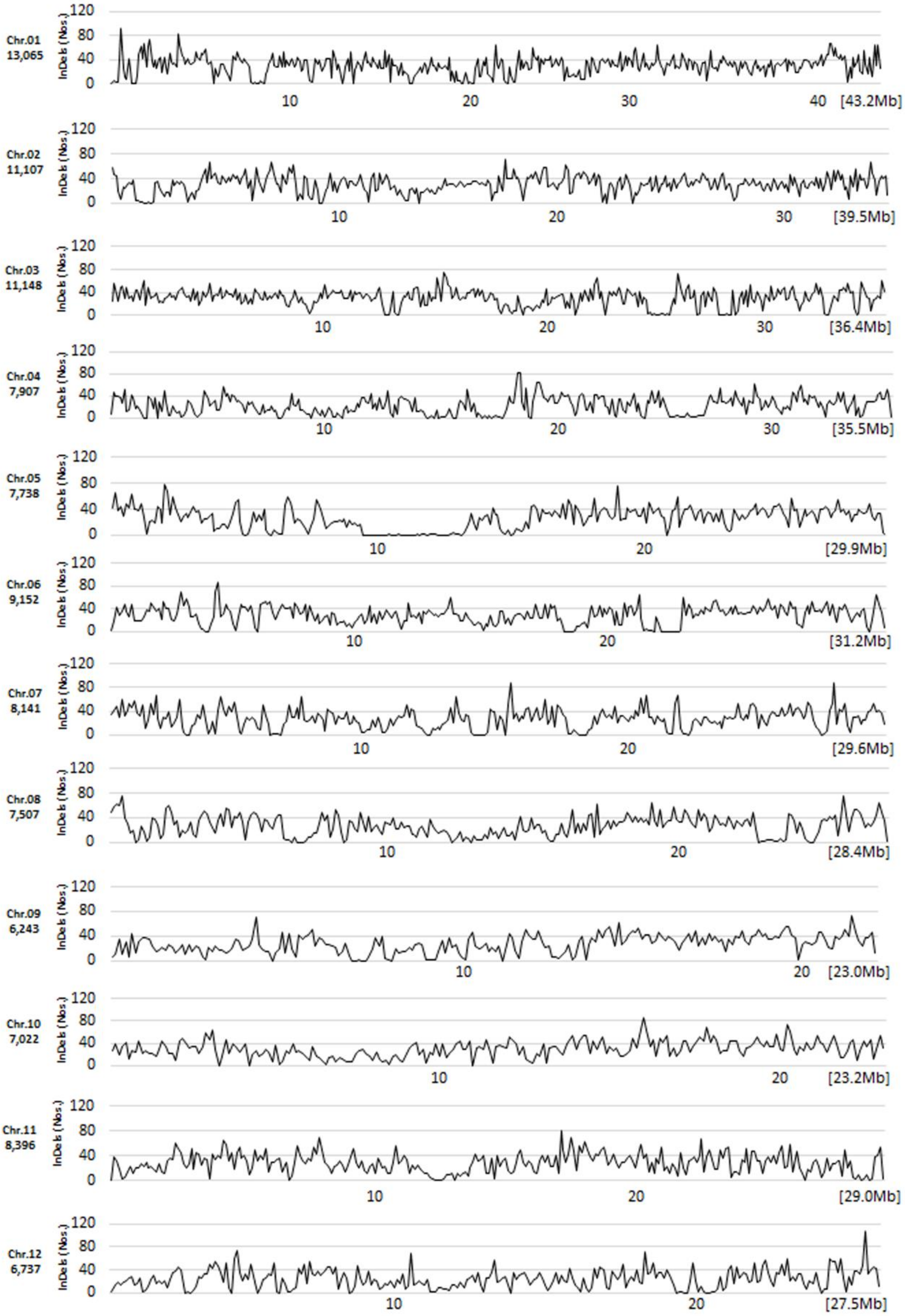

Supplementary Fig. S2: Distribution of InDels detected in Swarna in each chromosome. The x-axis represented the physical distance along each chromosome, split into 100 kb windows. The total size of each chromosome was shown in brackets. The y-axis indicated the number of SNPs. The total number of InDels in each chromosome was mentioned.

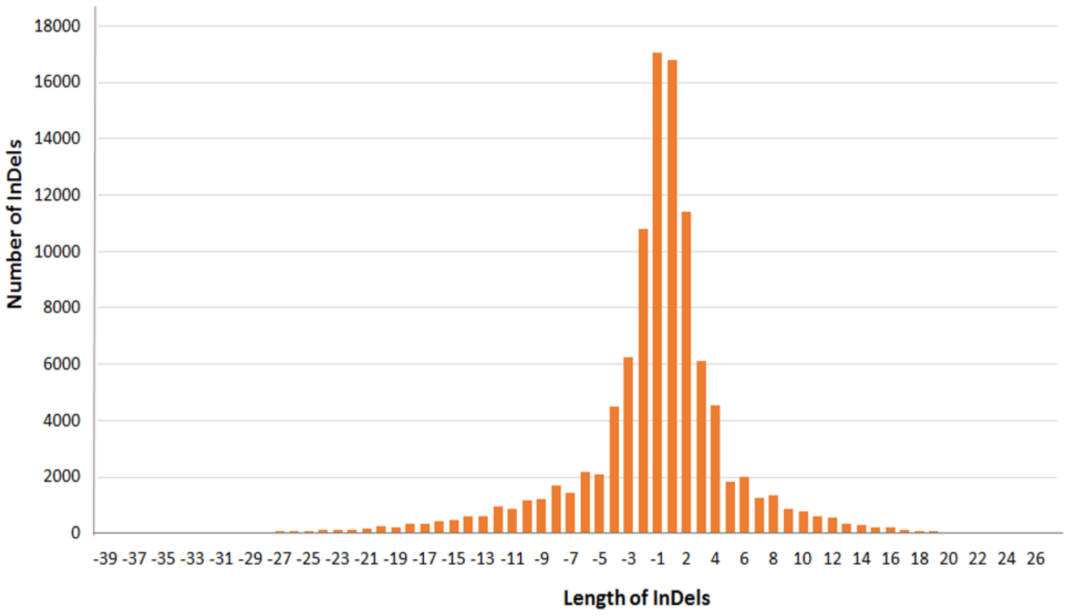

Supplementary Fig. S3: Distribution of the length of InDels. X-axis represents the length of InDels. The Y-axis shows the number of InDels in each length.

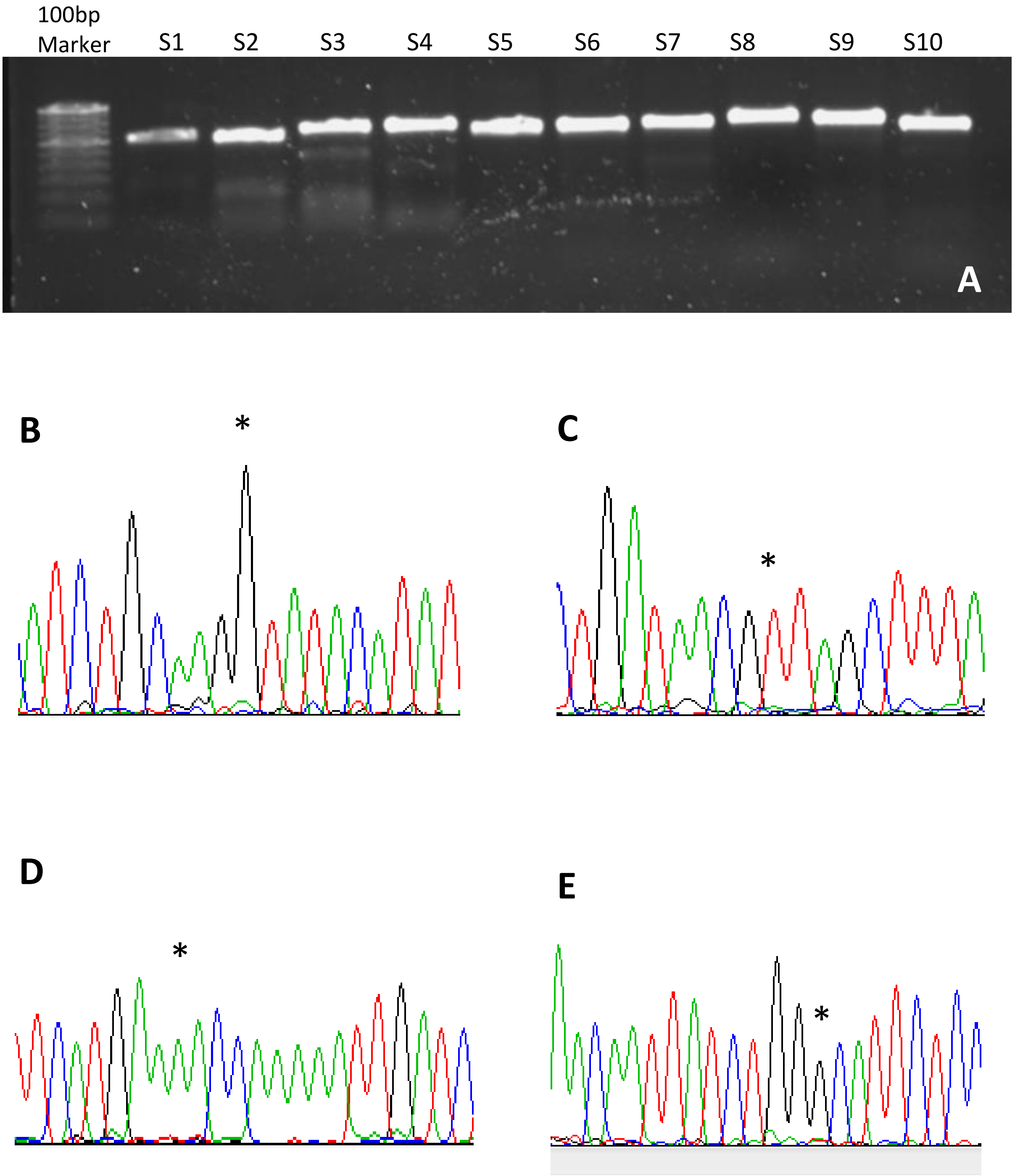

Supplementary Fig. S4: Validation of SNPs in SSRG genes by Sanger sequencing. Amplification of 10 target regions (S1 to S10) by PCR amplification (A), and validation of T/G SNP at position 1,765,761 (B), C/T at position 5,140,824 (C), C/A at position 31,758,208 (D), and A/G at position 12,918,525 (E). * indicates the position of SNP.
